# Racial Disparities in Clinical and Cost Outcomes Among Heart Transplant and Left Ventricular Assist Device Recipients: A Systematic Review

**DOI:** 10.7759/cureus.94135

**Published:** 2025-10-08

**Authors:** Abdelrahman A Bayoumi, Hamdi Lababidi, Karam L Khasawneh, Parth Bhagat, Amar M Ghaleb, Christopher Bobier, Beth Bailey

**Affiliations:** 1 School of Medicine, Central Michigan University College of Medicine, Mount Pleasant, USA; 2 Health Services Research, Central Michigan University College of Medicine, Mount Pleasant, USA

**Keywords:** access to health care, clinical outcomes, cost outcomes, heart failure, heart transplant, left ventricular assist device (lvad), racial and ethnic disparities

## Abstract

Although racial disparities in cardiac interventions have been studied extensively, disparities in heart transplants (HTs) and left ventricular assist devices (LVADs) have not been well-established. A systematic review was undertaken wherein 20 studies were identified for investigation, with a total of 785,486 patients. Of these, 104,356 patients were studied to assess HT and LVAD access, 332,885 patients were studied to assess in-hospital mortality rate, 346,303 patients were studied to assess organ function, and 1,942 patients were studied to assess costs associated with HT or LVAD treatment among different racial groups. Access was evaluated via the percentage of patient race within each study's population. White patients received the majority of LVADs (44,248 patients, 63.34%) and HTs (21,273 patients, 61.67%), followed by Black patients for LVADs (16,437 patients, 23.53%) and HTs (7,603 patients, 22.04%). Hispanic/Latino patients had the lowest rates of LVADs (4,071 patients, 5.83%) and HTs (3,153 patients, 9.14%). Asian patients had the highest in-hospital mortality (4,104 patients, 39.2%), whereas White patients had the lowest (84,977 patients, 36.2%). Results of cost outcomes varied, with Asian patients having the highest mean cost and Black patients having the lowest mean cost for transplantation. Racial disparities in HTs and LVADs were noted, highlighting the need for multi-faceted approaches to provide equitable access and improved outcomes.

## Introduction and background

For patients with advanced heart failure (HF), heart transplant (HT) and left ventricular assist device (LVAD) placement are critical interventions, which significantly improve survival and quality of life [1]. However, longstanding demographic disparities in healthcare continue to affect both access to these treatments and patient outcomes. Studies have shown that race, socioeconomic status, geographic location, and other factors influence key aspects of the treatment process, including waitlist placement, donor organ allocation, and post-transplant survival [2,3]. These disparities contribute to inequities in life-saving care and challenge the fundamental principle of fairness in healthcare delivery. A focused examination of how racial disparities intersect with HF treatment can help identify current disparities in clinical outcomes, access, and financial burden. 

Prior research has consistently documented racial disparities in HT outcomes. For example, a study found that five-year mortality was 35.7% among Black HT recipients versus 26.5% among White recipients, with Black patients more likely to experience graft failure or die from cardiovascular causes [4]. These differences in mortality reflect broader inequities in access to life-saving interventions, even among patients with similar clinical indications. Despite being equally eligible for HT or LVAD treatment, Black and Hispanic patients with HF and reduced ejection fraction are less likely to receive HT or LVAD compared to White patients [5]. These disparities have narrowed over the years, but financial barriers present another significant concern. 

Hispanic American and African American recipients are more likely to be uninsured or reliant on government-sponsored programs such as Medicaid or Medicare, which often provide less comprehensive coverage than private insurance [6]. This disparity in coverage may contribute to differences in treatment accessibility and clinical decision-making, restricting opportunities for minority patients to receive HF interventions. Moreover, research indicates that even among patients receiving care at specialized HF and ventricular assist device (VAD) centers, Black patients have lower rates of VAD and HT utilization than White patients, even after adjusting for disease severity, quality of life, and patient preferences [3]. These findings suggest that systemic barriers continue to shape treatment decisions and perpetuate health inequities. Given the critical role of HT and LVAD in improving both survival and long-term outcomes in advanced HF, addressing these disparities is essential to ensuring equitable access to care. 

In this systematic review, we evaluated articles published between 1990 and 2024 to examine HT/LVAD placement outcomes and analyzed the distribution of resources by race. Specifically, we assess inequities in treatment access, clinical outcomes, and cost-related burdens associated with these interventions. By analyzing these disparities, this review aims to highlight critical gaps in healthcare equity and inform strategies for reducing racial disparities in HF treatment. 

## Review

Materials and methods

Search Strategy and Selection Criteria 

Preferred Reporting Items for Systematic Reviews and Meta-Analyses (PRISMA) guidelines were followed. A search strategy was developed by combining key terms and medical subject headings (MeSH) related to outcomes of patients who received HTs and LVADs. Electronic database searches were performed on PubMed, Scopus, and CINAHL (Cumulative Index to Nursing and Allied Health Literature) Plus. The keywords used in our systematic search include “Racial Disparities”, “Heart Failure”, “Clinical Outcomes”, “Left Ventricular Assist Device”, “Heart Transplant”, and “Cost Outcomes.” All publications from 1990 to 2024 were searched by one reviewer with a restriction to articles written in English. We selected 1990 as the start date due to the advancements and improved outcomes of both HT and LVAD placement associated with that time. 

Records identified were imported into Rayyan for deduplication [7]. Each study was manually screened by at least two authors. Inclusion criteria were human/clinical research, peer-reviewed studies, HT or LVAD receipt, and presence of any outcome of interest. Outcomes of interest included survival, quality of life, graft function (HT or LVAD monitoring via tests/imaging), organ function (early post-operative monitoring via blood tests, electrocardiogram, etc), functional capacity (mid-term monitoring via cardiac output, left ventricular ejection fraction, etc), and cost outcomes. Exclusion criteria were reviews/meta-analyses, case reports, case series, book chapters, clinical guidelines, and study of any patients <18 years of age. No ongoing studies were searched or used in the investigation process. Further full-text review of articles was then performed. Articles were excluded if full texts were inaccessible or if data on racial disparities were unavailable. Any disagreements about the inclusion of articles were resolved by discussion. 

**Figure 1:**
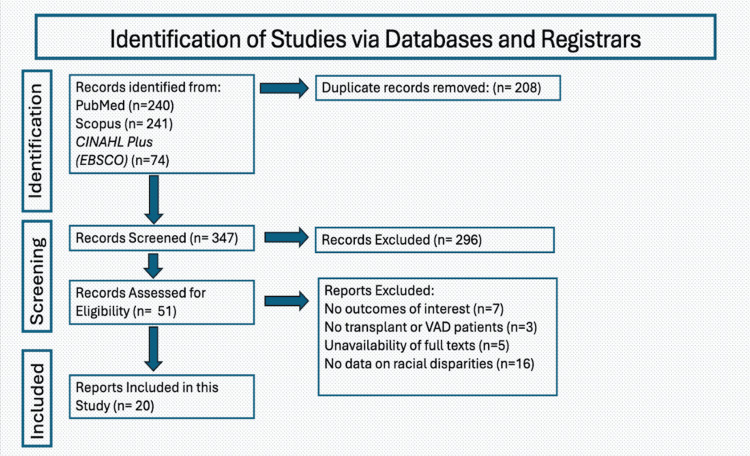
PRISMA flowchart for study selection PRISMA: Preferred Reporting Items for Systematic Reviews and Meta-Analyses

The search strategy identified 347 unique records, of which 296 were excluded during the initial abstract screening. The remaining 51 articles were moved forward for an in-depth full-text review. This resulted in another 31 articles being excluded. Out of the 31 excluded articles, seven were excluded for not having outcomes of interest, three were excluded for not having any transplant or VAD patients, five were excluded for unavailability of the full text, and 16 were excluded for not having data on racial disparities. There were a final 20 articles remaining in our review [8-27]. Figure 1 shows the PRISMA flowchart.

Data Extraction and Statistics

Each study was assessed for the percentage of LVAD and HT access by race. These percentages were either calculated or reported from studies directly. If studies pooled access data among various cardiac interventions wherein data specific to LVADs or HTs was not possible to obtain (such as both left and right VADs), then access data for these studies was not reported. 

LVAD and HT access percentages from individual studies were reported. In addition, data were pooled to form aggregate LVAD access as well as aggregate HT access percentages. The raw number of patients from each relevant study belonging to race categories was combined and divided by the grand total number of patients to derive these aggregate access percentages. If studies reported LVAD or HT access data for some races but not others, then access data from these studies were still reported, but these studies were excluded from calculations of aggregate access percentages to avoid over- or under-representing any race. Weighted samples, as reported in individual studies, were used preferentially over unweighted samples. 

Data on in-hospital mortality, quality of life, graft function, organ function, and functional capacity were also assessed. Percentages were considered the preferred statistic to maintain consistency with the access variable, although additional quantitative data were considered. Cost outcomes were assessed as well. 

Bias/Quality Assessment

We assessed risk of bias using validated tools according to study design: Newcastle-Ottawa Scale (NOS) for cohort studies [28] and the Appraisal tool for Cross-Sectional Studies (AXIS) tool for cross-sectional studies [29]. Narrative reviews and clinical guidelines were excluded from risk of bias assessment. The results are presented in Tables 1, 2.

**Table 1:** Newcastle–Ottawa Scale (NOS) quality assessment of cohort studies The NOS tool was applied to evaluate the quality of included cohort studies across three domains: selection, comparability, and outcome. A score of 7-9 indicates low risk of bias, a score of 4-6 indicates moderate risk of bias, and a score of <4 indicates high risk of bias [28].

Study (author, year)	Design	Tool	Selection (0–4)	Comparability (0–2)	Outcome/Exposure (0–3)	Total (0–9)	Overall Risk
Syed et al., 2022 [8]	Retrospective cohort	NOS	3	1	1	5	Moderate
Hernandez et al., 2007 [9]	Retrospective cohort	NOS	3	1	1	5	Moderate
Kilic et al., 2013 [10]	Retrospective cohort	NOS	3	1	2	6	Moderate
Liu et al., 2020 [11]	Retrospective cohort	NOS	3	1	1	5	Moderate
Ueyama et al., 2020 [12]	Retrospective cohort	NOS	3	1	1	5	Moderate
Vallabhajosyula et al., 2020 [13]	Retrospective cohort	NOS	3	1	1	5	Moderate
Shah et al., 2019 [14]	Retrospective cohort	NOS	3	1	2	6	Moderate
Breathett et al., 2020 [16]	Retrospective cohort	NOS	3	2	1	6	Moderate
Vallabhajosyula et al., 2020 [17]	Retrospective cohort	NOS	3	1	1	5	Moderate
Kim et al., 2020 [18]	Retrospective cohort	NOS	3	1	1	5	Moderate
Ahmed et al., 2020 [19]	Retrospective cohort	NOS	3	1	1	5	Moderate
Shah et al., 2020 [20]	Retrospective cohort	NOS	3	1	1	5	Moderate
Al-Ani et al., 2020 [21]	Retrospective cohort	NOS	3	1	1	5	Moderate
Gioli-Pereira et al., 2019 [22]	Prospective cohort	NOS	4	1	3	8	Low
Minhas et al., 2023 [23]	Retrospective cohort	NOS	3	1	1	5	Moderate
Ismail et al., 2024 [24]	Retrospective cohort	NOS	3	1	1	5	Moderate
Isath et al., 2022 [25]	Retrospective cohort	NOS	3	1	1	5	Moderate
Dewaswala et al., 2024 [26]	Retrospective cohort	NOS	3	1	1	5	Moderate
Singh et al., 2011 [27]	Retrospective cohort	NOS	4	1	3	8	Low

**Table 2:** AXIS tool quality assessment of cross-sectional studies Cross-sectional studies included in our review were evaluated using the AXIS tool: sampling (Adequate = representative sample), measurement (Appropriate = valid/reliable methods), bias consideration (Partial = some but not all biases addressed), and ethics (Reported = ethical approval/consent stated). An overall risk rating (low, moderate, high) was assigned based on domain ratings [29]. AXIS: Appraisal tool for Cross-Sectional Studies

Study (author, year)	Design	Tool	Sampling	Measurement	Bias Consideration	Ethics	Overall Risk
Roman et al., 2016 [15]	Cross-sectional	AXIS	Adequate	Appropriate	Partial	Reported	Moderate

Results

Overview of Included Studies

Table 3 summarizes the studies included in this review, detailing their design, sample size, study population, and key findings. This overview enables a clear comparison of study characteristics and outcomes across the literature. Notably, while each study reports its total enrolled population, only participants meeting our inclusion criteria were included in this review, so the overall sample size included in our review is smaller than the sum of individual study populations.

**Table 3:** Overview of included studies Columns summarize the reference, study design, total sample size, study population, and key findings. Sample size reflects the number of participants enrolled in each study; however, the total sample size for this review differs because only participants meeting our inclusion criteria contributed to the data. ECMO: extracorporeal membrane oxygenation; LVAD: left ventricular assist device; MCS: mechanical circulatory support; LOS: length of stay; PCI: percutaneous coronary intervention; HT: heart transplant

Reference	Study Design	Sample Size	Study Population	Key Findings
Syed et al., 2022 [8]	Retrospective cohort	1,633,877	National Inpatient Sample, United States	Black patients had lower utilization of ECMO; racial differences noted in outcomes.
Hernandez et al., 2007 [9]	Retrospective cohort	5,735	Post-cardiac surgery patients, United States	Black patients less likely to receive durable LVADs; worse early outcomes.
Kilic et al., 2013 [10]	Retrospective cohort	4,259	National Inpatient Sample, United States	Elderly and minority patients less likely to undergo LVAD implantation.
Liu et al., 2020 [11]	Retrospective cohort	184	Multicenter registry, United States	Racial disparities in renal replacement after LVAD implantation.
Ueyama et al., 2020 [12]	Retrospective cohort	27,132	Inpatient sample, United States	Black patients had higher in-hospital mortality after LVAD.
Vallabhajosyula et al., 2020 [13]	Retrospective cohort	402,825	Nationwide Inpatient Sample, United States	Black/Hispanic patients less likely to receive MCS; worse outcomes.
Shah et al., 2019 [14]	Retrospective cohort	7,046	United States registry	Infection and stroke risk varied by race; Black patients had higher complication rates.
Roman et al., 2016 [15]	Cross-sectional	956	Neuropsychological testing cohort, United States	Minority candidates had lower baseline cognitive scores, potentially affecting candidacy.
Breathett et al., 2020 [16]	Retrospective cohort	1,942	Medicaid expansion vs non-expansion states, United States	Expansion improved LVAD use in Black patients but disparities persisted.
Vallabhajosyula et al., 2020 [17]	Retrospective cohort	168,645	Nationwide, United States	Minorities less likely to receive percutaneous LVAD vs IABP; worse outcomes.
Kim et al., 2020 [18]	Retrospective cohort	332,885	United States hospitals	Black and Hispanic patients had lower MCS utilization.
Ahmed et al., 2020 [19]	Retrospective cohort	3,511	National Inpatient Sample, United States	Minority patients had longer LOS; gender and race influenced outcomes.
Shah et al., 2020 [20]	Retrospective cohort	1,531	Registry, United States	Minority HT recipients had worse PCI outcomes.
Al-Ani et al., 2020 [21]	Retrospective cohort	580	ED utilization cohort, United States	Minority patients had higher ED utilization and post-LVAD readmissions.
Gioli-Pereira et al., 2019 [22]	Prospective cohort	695	Brazil, a middle-income country	Socioeconomic disparities (income/insurance) drove outcomes more than race.
Minhas et al., 2023 [23]	Retrospective cohort	30,585	Nationwide Inpatient Sample, United States	Lower socioeconomic status associated with worse outcomes regardless of race.
Ismail et al., 2024 [24]	Retrospective cohort	110,015	National Inpatient Sample, United States	Black and Hispanic patients less likely to undergo HT; higher in-hospital mortality.
Isath et al., 2022 [25]	Retrospective cohort	1,953	National Inpatient Sample, United States	Sudden cardiac arrest post-HT more common in minority groups.
Dewaswala et al., 2024 [26]	Retrospective cohort	20,180	National Inpatient Sample, United States	Sex and race interacted; minorities had worse transplant outcomes.
Singh et al., 2011 [27]	Retrospective cohort	160	United States registry	Long-term survival improved overall, but racial disparities persisted.

LVAD and HT Access 

**Figure 2:**
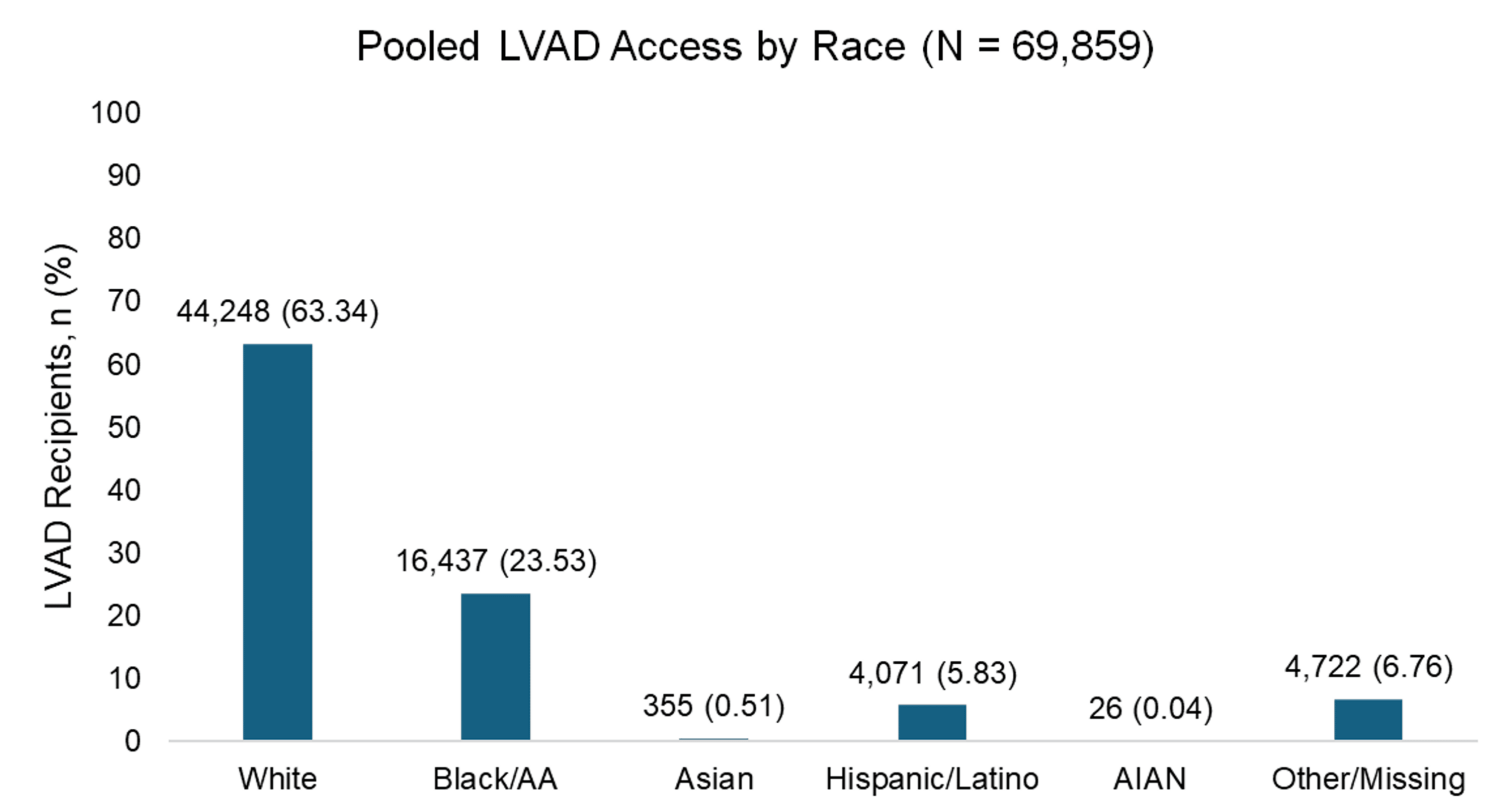
Pooled LVAD access by race Pooled percentages of LVAD access by race across the included studies [9-12], [17-19], and [21,23]. Values represent the weighted proportion of patients in each racial/ethnic category. LVAD: left ventricular assist device; AIAN: American Indian or Alaska Native

**Figure 3:**
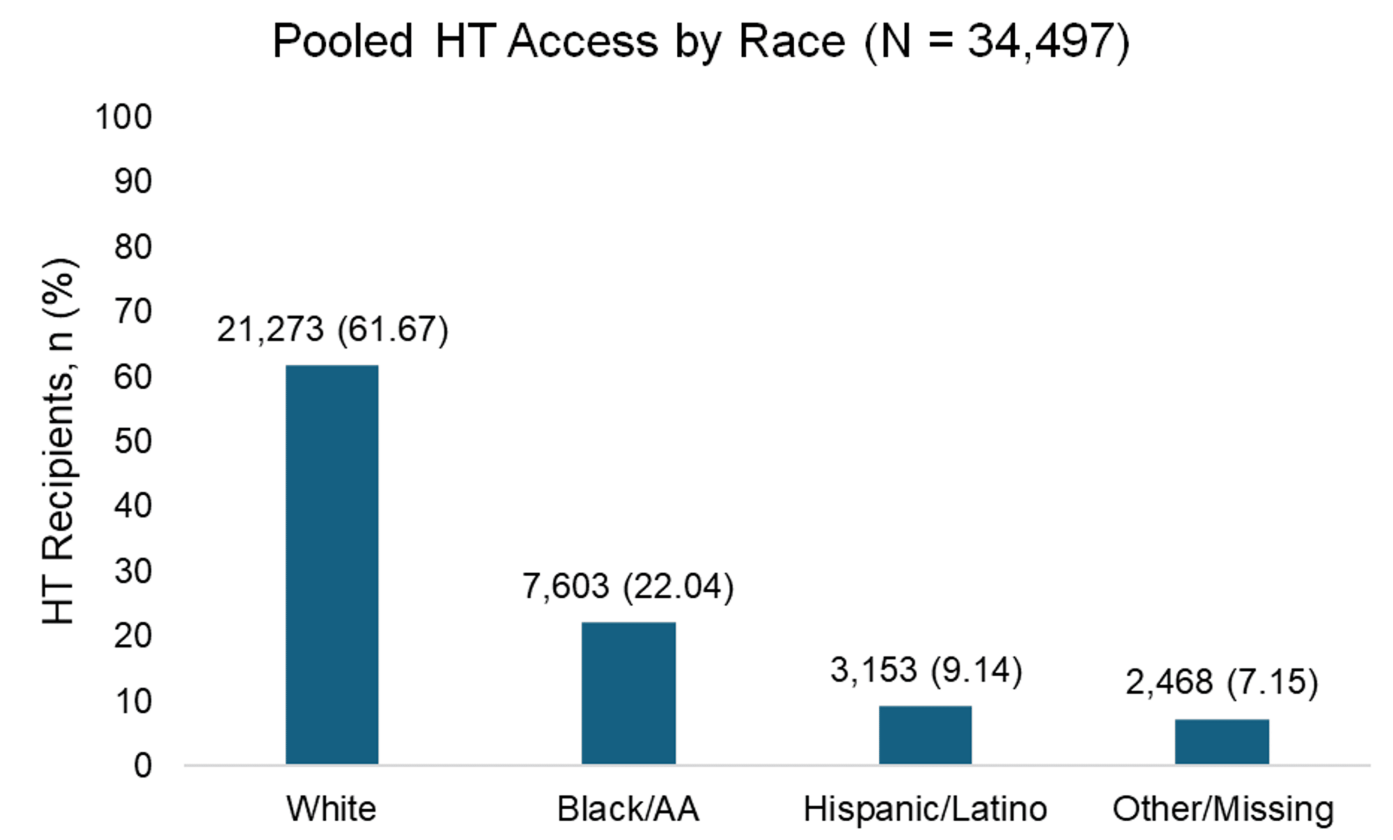
Pooled HT access by race Pooled percentages of HT access by race across the included studies [23,24] and [20,26]. Values represent the weighted proportion of patients in each racial/ethnic category. HT: heart transplant

Our analysis revealed clear racial disparities in the utilization of advanced HF therapies. White patients consistently accounted for the highest proportion of both LVAD implantation and HT, while Black patients received these therapies at comparatively lower rates. Notably, other racial groups demonstrated even lower rates of access across both treatment modalities (Figures 2, 3).

Survival 

**Figure 4:**
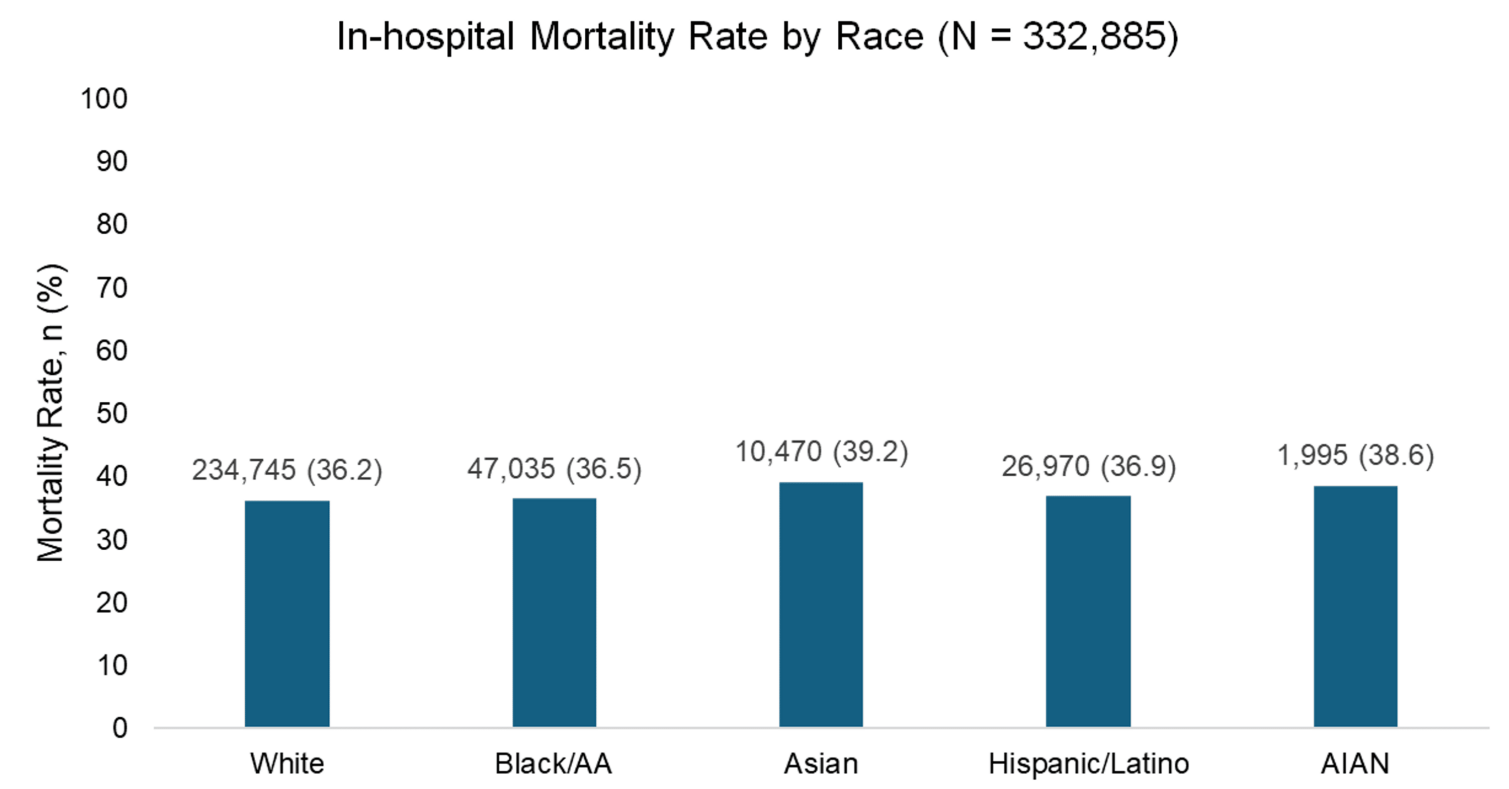
In-hospital mortality rate by race following mechanical circulatory support due to heart failure-induced cardiogenic shock Individual sample sizes represent the total number of individuals in each race. Percentages indicate the proportion of individuals in each race category that experienced in-hospital mortality across the studies included [18].

Our analysis showed variations in in-hospital mortality rates by race among individuals receiving mechanical circulatory support (MCS) due to HF-induced cardiogenic shock (Figure 4). Among the racial groups analyzed, individuals of Asian descent exhibited the highest in-hospital mortality rate, while White individuals demonstrated the lowest in-hospital mortality rates. 

*Organ Function * 

**Figure 5:**
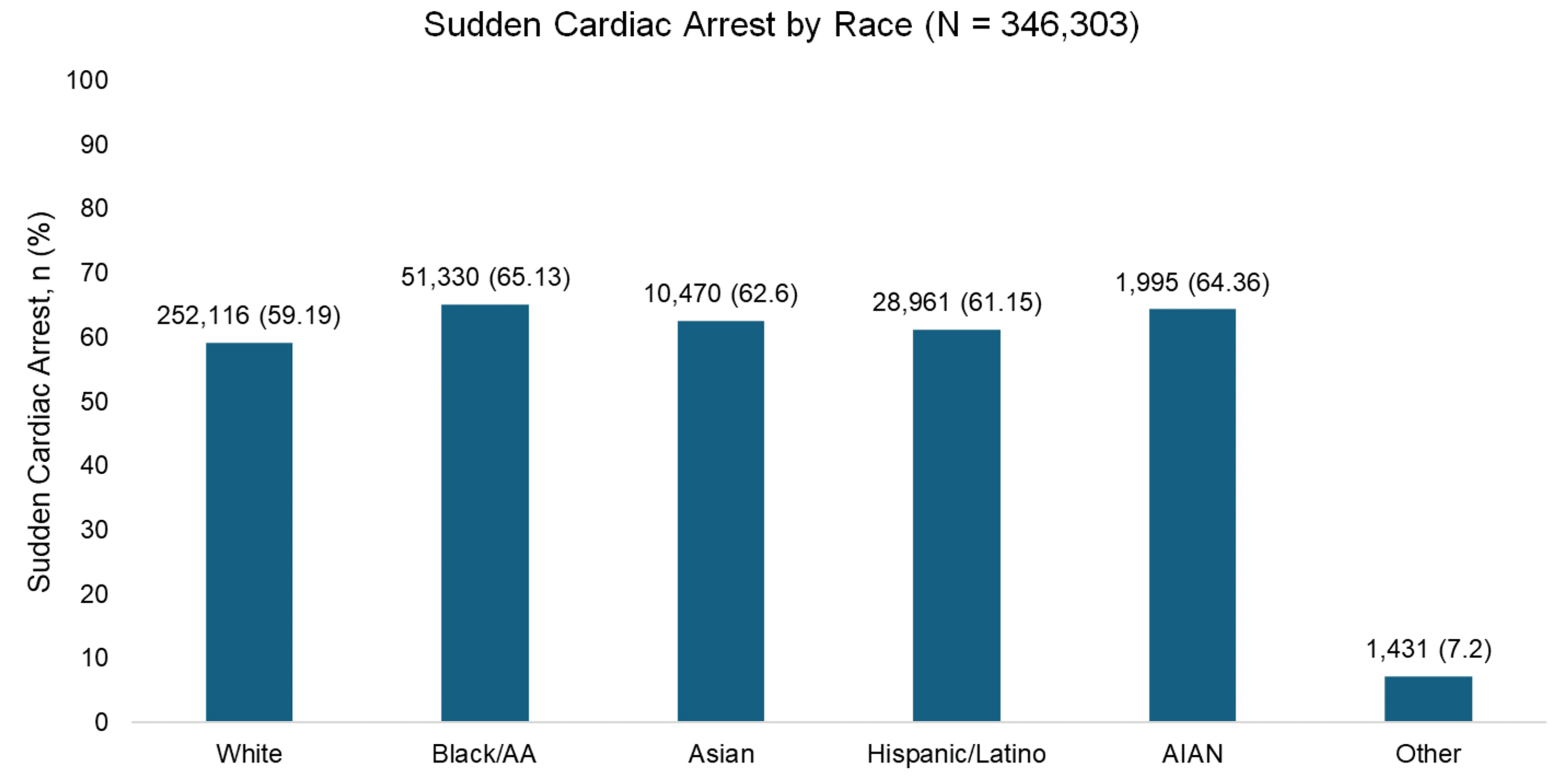
Pooled organ function by race Individual sample sizes were pooled across the studies [12-14] and [8,18,25,27] and represent the total number of individuals in each race. Percentages indicate the proportion of individuals in each race category that experienced sudden cardiac death. Data in the “Other” category was decided by individual studies. Depending on the study, it may have included any of the other race categories and/or additional race categories. AA: African American; AIAN: American Indian or Alaska Native

Of the studies analyzed, several included data on organ function as measured by the incidence of sudden cardiac arrest across different racial and ethnic groups. When pooled together, these data showed variation in the proportion of individuals who experienced sudden cardiac arrest by race (Figure 5). Among the 346,303 individuals included, the rates of sudden cardiac arrest were 59.19% in White patients, 65.13% in Black/African American patients, 62.6% in Asian patients, 61.15% in Hispanic/Latino patients, and 64.36% in American Indian/Alaska Native patients, with 7.2% categorized as “Other.” While differences were observed numerically, most studies did not report statistically significant disparities across races in the incidence of sudden cardiac arrest following heart transplantation or LVAD insertion.

*Cost Outcomes * 

**Figure 6:**
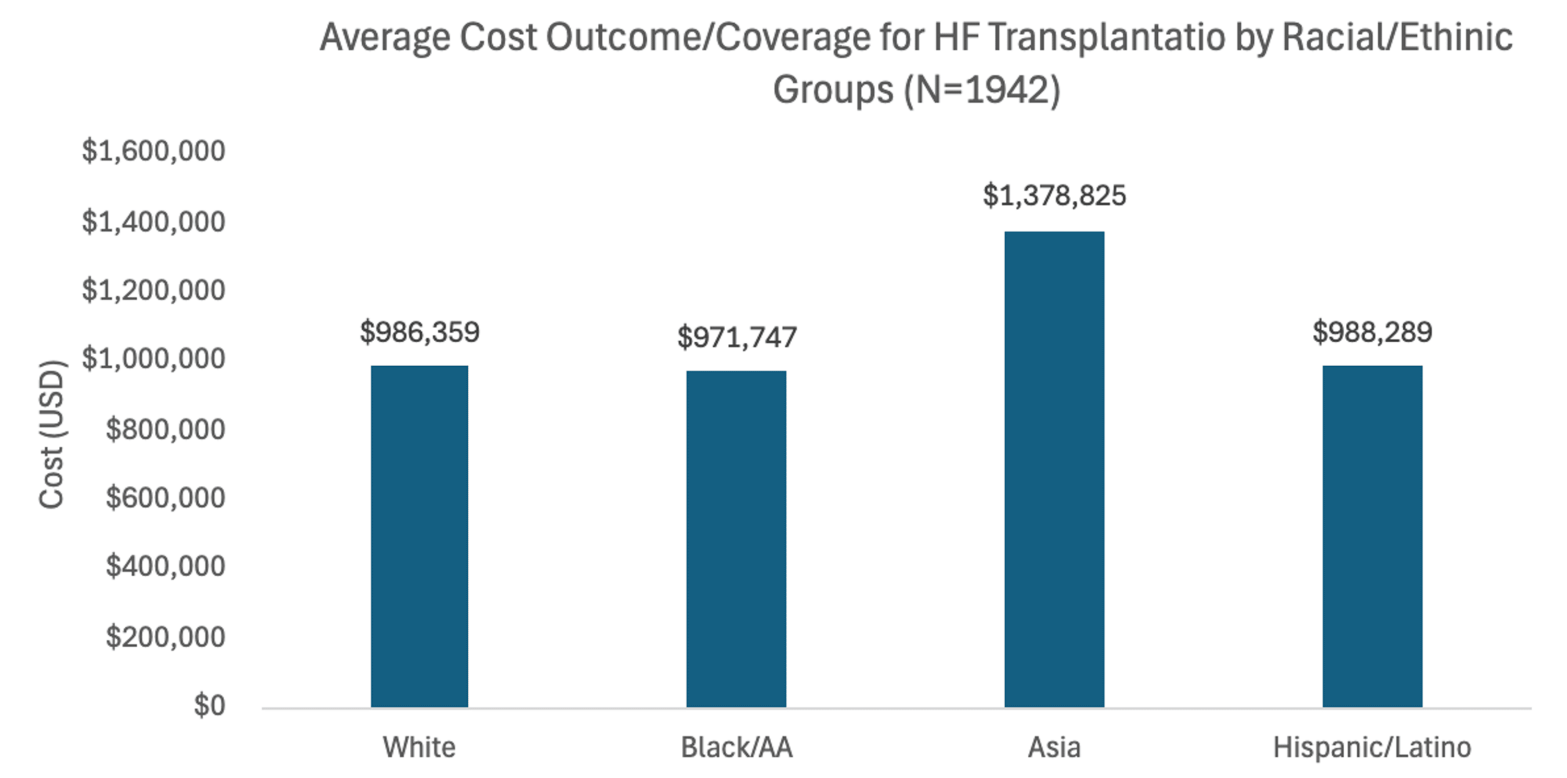
Average cost outcome for HF transplantation across different ethnic groups Average cost outcome/coverage for HF transplantation across different ethnic and racial groups. The figure depicts the mean charges applied to different racial groups with heart transplant receiving heart transplantation [16]. AA: African American

Of the studies reported in our analysis, only one study highlighted significant disparities in coast outcomes [16]. The study revealed that among individuals with HF undergoing HT, those of Asian descent incurred the highest mean charges, whereas Black and African American individuals had the lowest mean charges (Figure 6).

Quality of Life, Graft Function, Functional Capacity 

Although studies were included in the review if the quality of life, graft function, and functional capacity outcomes were discussed, there was no quantitative data reported by any study discussing these outcomes. 

Discussion

Our systematic review revealed significant racial disparities in both access to and outcomes of heart transplantation and LVADs. The findings demonstrate persistent inequities across multiple domains, including treatment access, mortality rates, and financial burden. 

Access Disparities 

The pooled data analysis revealed that White patients consistently received the majority of both LVAD and HT interventions, while Black and African American patients received substantially lower proportions. This disparity is particularly concerning given the higher prevalence and earlier onset of HF among Black and African American individuals. Hispanic/Latino patients represented only 5.83% of LVAD recipients and 9.14% of HT recipients, while Asian and American Indian/Alaska Native patients had extremely limited representation in both interventions. These findings align with previous research demonstrating persistent racial disparities in advanced HF therapies and suggest that, despite increased awareness, significant barriers to access remain. 

Previous studies on HT and LVAD utilization have consistently reported higher procedure rates among White men compared to Black and Hispanic/Latino men. However, recent trends indicate a gradual increase in the adoption of both HT and LVAD interventions across all three racial groups. This shift may reflect the impact of historical disparities in access, where structural inequities have influenced baseline measurements and the allocation of advanced HF therapies. These findings warrant further investigation to determine whether recent efforts to address these disparities are sufficient to achieve equitable access for all racial and ethnic groups. 

Clinical Outcomes 

Our analysis of in-hospital mortality rates following MCS revealed concerning disparities, with Asian patients experiencing the highest mortality rates. This finding warrants further investigation into potential contributing factors, such as delayed presentation, differences in comorbidity profiles, or variations in care delivery. The observed differences in mortality rates across racial groups suggest the need for targeted interventions to improve outcomes for vulnerable populations. We expected a similar result as previous studies noted that although early post-transplant survival has improved equally in recent years, longer-term survival only improved in Whites but not in Black or Hispanic HT recipients. 

Financial Burden and Healthcare Coverage 

The cost analysis revealed substantial variations in financial burden across racial groups. Asian patients faced significantly higher mean charges compared to other racial groups, while Black and African American patients had the lowest mean charges. However, these figures must be interpreted in the context of access to financial support mechanisms. White and Black and African American patients received a higher percentage of fundraising support compared to Asian and Hispanic/Latino patients, suggesting potential disparities in access to financial resources and support networks. The literature suggests that factors like socioeconomic status, clinician bias, and immunologic differences contribute to these disparities. 

The analysis of Medicaid coverage revealed higher odds of coverage for Black and African American patients in both Affordable Care Act adopter and non-adopter states, possibly reflecting differences in socioeconomic status and insurance status across racial groups. This finding highlights the critical role of healthcare policy in addressing disparities in access to advanced HF therapies. 

Implications for Clinical Practice and Policy 

These findings have several important implications. Our research shows that healthcare systems need to implement targeted strategies to improve access to advanced HF therapies for underrepresented racial groups. Furthermore, clinical protocols should be reviewed to address potential biases in patient selection and care delivery that may contribute to disparate outcomes. Finally, financial support mechanisms need to be more equitably distributed across racial groups to ensure that cost barriers do not exacerbate existing disparities. 

Limitations 

Several limitations should be considered when interpreting these findings. The "other" category classification varied across studies, potentially affecting the accuracy of racial categorization. Secondly, some articles identified individuals within multiple racial categories, negatively affecting the precision of isolating racial differences. Thirdly, the retrospective nature of the included studies limits our ability to establish causality for the observed disparities. 

Future Research Directions 

Future research should focus on investigating the specific mechanisms underlying disparate mortality rates among different races compared to White patients. Furthermore, research should focus on examining the intersection of racial disparities with other social determinants of health while also assessing the impact of various healthcare policies on reducing racial disparities in access and outcomes. 

## Conclusions

Racial disparities in HT and LVAD therapy persist across various domains. Our findings suggest that addressing these disparities requires a multifaceted approach incorporating clinical practice changes, policy reforms, and continued research. Despite increased awareness and some policy changes, significant work remains to achieve equitable access and outcomes in advanced HF therapy. 
